# Unlocking India’s Potential in Managing Endocrine-Disrupting Chemicals (EDCs): Importance, Challenges, and Opportunities

**DOI:** 10.1007/s12403-022-00519-8

**Published:** 2022-12-12

**Authors:** Brij Mohan Sharma, Martin Scheringer, Paromita Chakraborty, Girija K. Bharat, Eirik Hovland Steindal, Leonardo Trasande, Luca Nizzetto

**Affiliations:** 1 Faculty of Science, RECETOX, Masaryk University, Kotlarska 2, 62500 Brno, Czech Republic; 2 Environmental Science and Technology Laboratory, Department of Chemical Engineering, SRM Institute of Science and Technology, Kattankulathur, Tamil Nadu 603203 India; 3 Mu Gamma Consultants Private Limited, Gurugram, Haryana India; 4 Norwegian Institute for Water Research (NIVA), Økernveien 94, 0579 Oslo, Norway; 5 Norwegian University of Life Sciences (NMBU), Universitetstunet 3, 1432 Ås, Norway; 6 Department of Pediatrics, Environmental Medicine, and Population Health, New York University Grossman School of Medicine, New York, NY USA; 7 NYU College of Global Public Health, New York, NY USA

**Keywords:** Endocrine-disrupting chemicals, Chemical management policy, Environmental governance reform, Human health, Developing countries, India

## Abstract

Endocrine-disrupting chemicals (EDCs) are a prime concern for the environment and health globally. Research shows that in developing countries such as India both the environment and human populations are severely exposed to EDCs and consequently experience rising incidents of adverse health effects such as diabetes and cancers. In this paper, we discuss the current EDC management approach in India, critically assess its limitations, and describe opportunities for potential improvements. Foremost, current EDC management actions and interventions in India are fragmented and outdated, and far behind the modern and comprehensive approaches adopted in the European Union and other developed countries. Strong and well-planned actions are required on various fronts of science, policy, commerce, and public engagement. These actions include the adoption of a dedicated and modern regulatory framework for managing EDCs, enhancing capacity and infrastructure for EDC monitoring in the environment and human population, employing public–private partnership programs for not only managing EDCs but also in the sectors that indirectly contribute toward the mismanagement of EDCs in the country, and raising awareness on EDCs and promoting health-preserving consumption habits among the public. As India hosts a large proportion of the global human population and biodiversity, the success or failure of its actions will substantially affect the direction of global efforts to manage EDCs and set an example for other developing countries.

## Introduction

Endocrine-disrupting chemicals (EDCs) are ubiquitous in the environment, human food, and consumer products (Wong and Durrani 2017; Vaccher et al. 2020; Ostad-Ali-Askari 2022). Thousands of chemicals have been found to have the potential to impair at least one of the three endocrine pathways (oestrogen, androgen, and thyroid) (Kahn et al. 2020), and the United Nations Environment Programme (UNEP) lists 45 chemicals as EDCs as the result of being screened through at least one “thorough scientific assessment” (UNEP 2017).

EDCs are responsible for adverse health outcomes that can emerge over the entire human lifespan (Johansson et al. 2017; Street et al. 2018; Kahn et al. 2020; Nesan and Kurrasch 2020). Susceptibility to EDC-induced conditions is not uniform across the world population, but is more pronounced among disadvantaged groups (Bornman et al. 2017; Ruiz et al. 2018b; Daniels et al. 2018; Attina et al. 2019; Quirós-Alcalá et al. 2020), especially those in developing countries in Sub-Saharan Africa (556 million), South Asia (533 million), and Latin America and the Caribbean (38 million). India alone accounts for 369 million multidimensionally poor people suffering from deprivation in health care, education, and living conditions (UNDP and OPHI 2020). Reducing EDC exposure and minimizing health consequences across the globe is paramount for achieving the Sustainable Development Goals (SDGs) (WHO 2016). This can be achieved by making chemicals- and health-related policies (and their implementation) in developing countries more effective, also by looking at what has and has not worked in industrialized countries.

While international harmonization of the scientific and technical approach to regulating and managing EDCs is a requirement, approaches should still be tailored to the local environment, political system, economy, and culture in developing countries. This paper describes and examines India’s current approach to managing EDCs, evaluates the existing management system’s strengths and limitations, and provides recommendations to endorse better public health protection from EDCs.

## Needs of EDC Management in Developing Countries: India’s Context

A large body of scientific evidence provides clear insights into the causal link between EDC exposure and health outcomes. Over the last two decades, Multilateral Environmental Agreements (MEAs) have emerged to protect human health and the environment from the adverse effects of particularly hazardous chemicals, such as Persistent Organic Pollutants (POPs) and Mercury. Even though these MEAs contingently encompass several EDCs, EDCs are not included as such (UNEP 2019; Escobar-Pemberthy and Ivanova 2020). The success of MEAs largely depends on how and to what extent their requirements are met in developing countries, particularly in those experiencing rapid economic and industrial transition, acting as significant emitters of hazardous chemicals (Evers et al. 2016).

India’s economy has grown tremendously in recent times (accounting for US$ 3.12 trillion in the financial year 2021–22) and with a heavy toll on the environment. The rapid economic transition and associated fast industrialization (particularly of the Indian chemical industry) and urbanization in India have triggered multifaceted environmental and social challenges including rising greenhouse gas emissions, depletion of natural resources, and surge in concentrations of various synthetic chemical contaminants in the atmosphere and water resources. In the last two decades, the cost of damage done by pollution and natural disasters in India has risen from $14 to $80 billion annually, close to 6% of the gross domestic product (GDP) (Managi and Jena 2008; The World Bank 2013). In the European Union (EU) and the United States (US), the estimates of the burden and disease cost of EDCs reached up to €163 million and $340 million, respectively, even though only a small subset of EDCs and associated health outcomes was taken into consideration (Kassotis et al. 2020). Similar calculations have not been made for India. However, fragmentary data on EDC levels in human and environmental samples in India suggest that pressure from these contaminants is similar to Europe and the US (Sharma et al. 2014a; Fång et al. 2015; Breivik et al. 2016; Katsikantami et al. 2016). Moreover, poverty, malnutrition, illiteracy, poor living conditions, and limited access to healthcare by a substantial fraction of the population are likely to make India more susceptible to severe social and health impacts of EDCs.

A review of data on prioritized EDCs (e.g. dichlorodiphenyltrichloroethane (DDT), lindane, polychlorinated biphenyls (PCBs), etc.) reported elevated concentrations in the Indian environment and human population (compared to the international context including emerging economies like China) (Sharma et al. 2014a). Similarly, a recent global survey measuring selected EDCs (in particular DDT) in breastmilk, carried out by WHO and UNEP, highlighted that Indian women and infants are among the most exposed in the world (Berg et al. 2017). A recent nationwide pilot study has reported the widespread occurrence of per- and polyfluoroalkyl substances (PFASs) in humans from different locations across India including those residing along the pristine areas of the Indian Himalayas (Ruan et al. 2019). Another study focusing on pregnant women and their offspring from Southern India detected phenolic EDCs including parabens, bisphenol-A (BPA), and triclosan, at concentrations comparable to those detected in populations from developed countries like Japan and Spain (Shekhar et al. 2017). Similarly, recent studies have reported widespread exposure to phthalates across different locations in India (Babu-Rajendran et al. 2018; Mukherjee Das et al. 2022). Furthermore, recent studies have also linked elevated EDC burden in the Indian population to adverse health effects such as childhood obesity, which is a major risk factor for other health issues including type 2 diabetes in later life stages (Xue et al. 2015; Dutta and Khadgawat 2015). However, all these pilot studies do not really provide a comprehensive assessment of EDC human exposure in India as compared to well-planned, systematic, and comprehensive national human biomonitoring (HBM) programs in Europe and North American countries. Nevertheless, one important conclusion from these studies is that the Indian population is exposed (in some cases to a similar degree as in developed countries) to both legacy pesticidal and industrial chemicals as well as modern emerging pollutants falling under the classifications of EDCs. Data on other groups of EDCs are scarce or entirely missing.

This hypothesis that in India there are increasing health impacts from EDC pressure on the human population is corroborated by data on diseases like diabetes and some cancers that have been linked to EDCs (Rachoń 2015; Lind and Lind 2018). Diabetes incidence in India increased from 26 million in 1990 to 65 million in 2016 compared to the population growth of about 50% during the same period (Pandey and Sharma 2018; Tandon et al. 2018). Similarly, the number of total deaths and disability-adjusted life-years (DALY) due to cancer doubled in India between 2009 and 2016 (Dhillon et al. 2018). Concurrently, there is documented evidence of increased EDC occurrence in both consumer products (reflecting global trends) and in the environment (Sharma et al. 2014a, 2021). Poor pollution control and chemical management combined with the rapidly changing socio-economic conditions in India certainly play a role in exacerbating negative impacts (Kaveeshwar and Cornwall 2014; Mallath et al. 2014; Sharma et al. 2014b; Dutta and Khadgawat 2015).

## Challenges for Managing EDCs in India

A scientifically informed assessment and management of risk is the main pillar of advanced chemical regulatory frameworks. In the EU, the regulation on Registration, Evaluation, Authorisation and Restriction of Chemicals (REACH) and the General Food Law (focused on the risk posed by plant protection product residues, artificial food additives, and contaminants in food) are examples of regulations that centralize risk analysis relevant also for EDCs and human health (Majcen 2017). These regulations consider risk assessment, risk management, and risk communication as three distinct, yet interdependent elements of the risk analysis framework whereby risk assessment is informed through a holistic analysis that considers all available data and knowledge (including technical data on the compounds, data on the usage of chemicals, knowledge, and data on their behaviour and fate during the life cycle, knowledge on exposure pathways and effects at a different level of biological organizations). Risk management is the set of measures adopted to effectively keep risks for humans and the environment under control. Finally, risk communication is essential for the effective implementation of risk management actions and involves all principal societal actors (industry and trade organizations, environmental and health organizations, customer associations, etc.). These regulations also introduce the precautionary principle in risk management as the norm to address any substances for which available data and knowledge are currently insufficient for drawing a robust assessment of risk.

EDC criteria are not yet fully embodied in the REACH and the General Food Law, mostly due to the challenge of short-listing substances for prioritization. However, the EU is currently making substantial progress in assimilating the latest research advances to define and apply EDC criteria for regulatory use. Efforts to systematically collect data on human biomonitoring of EDCs and associated health risks are ongoing, too (Choi et al. 2015; Apel et al. 2020).

Currently, India is updating its regulatory approach toward managing chemicals by drafting a new regulation, the Chemical (Management and Safety) Rules (CMSR). These rules are intended to replace two existing regulations—the Manufacture, Storage and Import of Hazardous Chemical Rules and the Chemical Accidents (Emergency Planning. Preparedness and Response) Rules. The CMSR aligns with the concept of the EU REACH regulation and is also termed “Indian REACH”, which requires manufacturers, importers, or authorized representatives to notify new or existing substances and register specific substances. Taking these EU regulations as a term of comparison, several challenges exist for India (and other rapidly industrializing developing countries) to lift EDC management and health protection to the state-of-the-art. These are discussed in the following subsections:

### EDC Management Overshadowed by Other Environmental and Public Health Emergencies

**Fig. 1:**
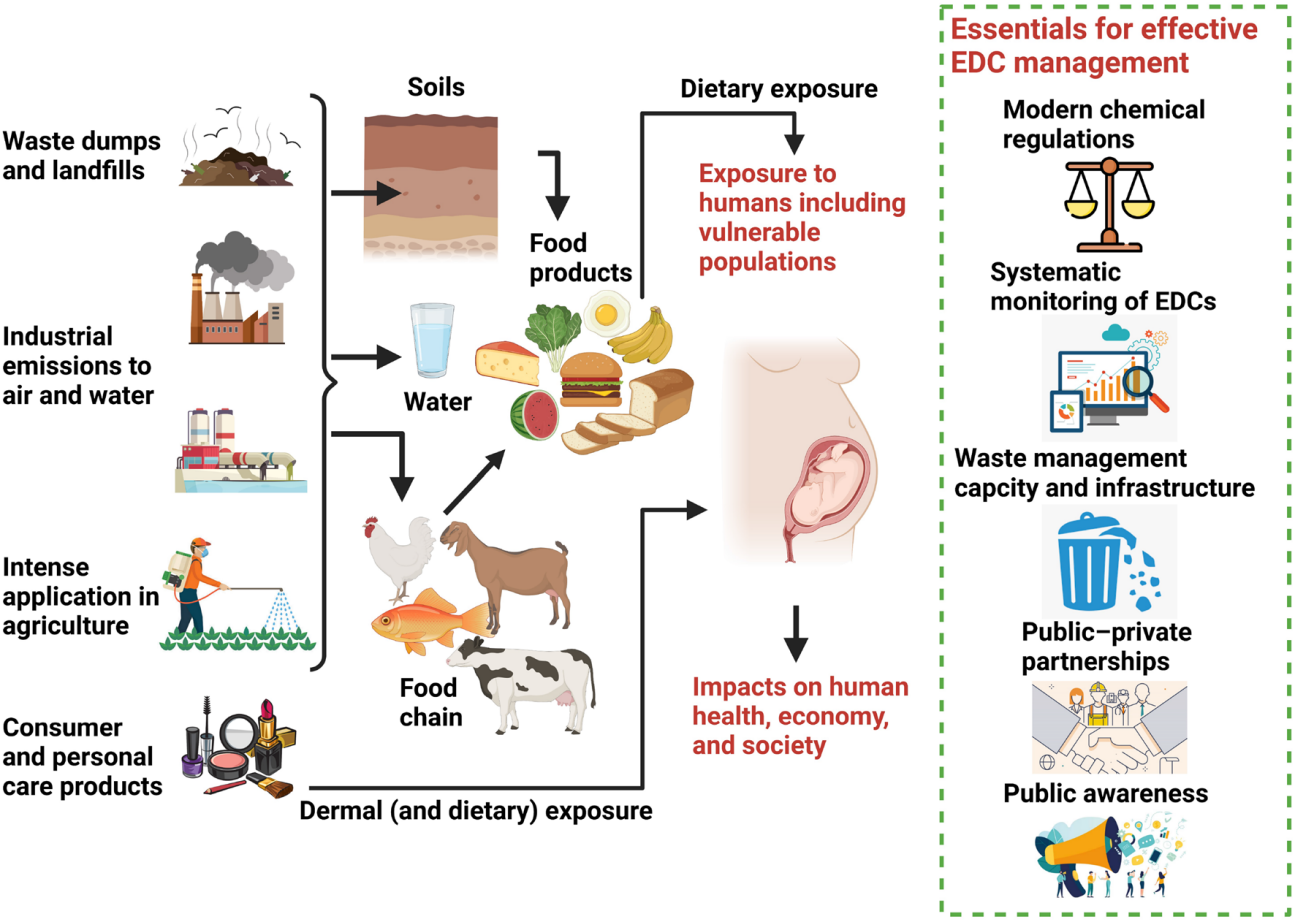
Major sources for EDCs exposure in the Indian population and the environment; and an overview of essential actions towards effective management of EDC pollution and exposure in India

India and many other developing countries are still facing multiple basic environmental and health issues. Implications of several underlying environmental and public health issues are more discernible and, as a result, they are prioritized over and receive more resources, infrastructure, and capacity for management than the very specific and technical issue of EDC pollution in many developing countries including India. The major recognized environmental and health challenges are air pollution, vehicular emissions, industrial emissions and wastes, water scarcity, sanitation, intensive mining activities, waste management (urban, rural, and biomedical), vector-borne diseases, child mortality and morbidity, hunger, malnutrition, etc. (Chandra 2015; Narain 2016; Ostad-Ali-Askari et al. 2017). In addition, open burning of dumped waste is a major source of atmospheric emission of several EDCs all over India (Chakraborty et al. 2021a) (Fig. 1). Air pollution in India has been associated with 1.67 million deaths (17% of the total deaths in the country) in 2019 (Pandey et al. 2021). Approximately 91 million people in India lack safe access to clean water (Water.org 2021). These challenges are exacerbated by other issues such as climate-change-driven environmental disasters, COVID-19 pandemic, and widespread poverty. India is among the top-ten countries in terms of absolute monetary damages caused by natural disasters, accounting for an average annual economic loss of $2.9 billion from 1990 to 2015 (Panwar and Sen 2020). A pre-COVID-19 estimate suggests that climate change-driven events can push 50 million Indians into poverty (Hallegatte et al. 2016). At the same time, these fundamental environmental and health issues overburden existing mechanisms and resources for handling EDCs in the country. For instance, the COVID-19 pandemic caused unexpected changes in the waste composition and constraints to the capacity of waste-pickers and informal waste collectors in manual sorting and recycling of waste (Sharma et al. 2020). Additionally, the COVID-19 crisis set off reverse migration from urban to rural areas within the country (United Nations Environment Programme 2020; Ranjan 2021), fostering populations to turn back to farming for both food and income, which may drive high pesticide consumption to meet the expectation of higher crop yield. Moreover, sewage treatment plants and wastewater treatment plants in India are non-functional at several places leading to direct discharge of waste and wastewater into the water bodies leading to EDC contaminations (Chakraborty et al. 2021b; Ostad-Ali-Askari and Shayannejad 2021). Apart from prime environmental and health issues, rapid expansion of megacities in India has resulted in a complete mismatch between waste production and processing capacity, for both municipal solid waste and industrial hazardous waste (Dutta and Jinsart 2020), ultimately contributing to burgeoning release of EDCs to the environment and their mismanagement. Expanding megacities further contribute to EDC pollution and human exposure by altering the food-supply chain and causing a shift from traditional, not chemical intensive, to modern and chemical-intensive food production, processing, and consumption system in developing countries like India (FAO 2019). Furthermore, the modern lifestyle in megacities directly exposes humans to EDCs including those present in consumer and personal care product and household products such as carpets, flooring, cleaning products, etc.

### Scarce or Unavailable Data from Monitoring, Trade, and Stockpiles of EDCs

Despite the evidence of increased exposure to EDCs, particularly to those used extensively in the past (Sharma et al. 2014b), there is no systematic and comprehensive National (or States-wide) HBM program in India. Available data are fragmented and primarily focus on pollution hotspots, which may disguise the actual EDC human exposure situation in the country (Sharma et al. 2014a). Reported EDCs in HBM studies from India typically include legacy pesticides (e.g. dichlorodiphenyltrichloroethane (DDT), hexachlorocyclohexane (HCH), industrial chemicals (e.g. polychlorinated biphenyl (PCB), solvents and heavy metals (Sharma et al. 2021). In contrast, data on a large set of extensively used EDCs including BPA, phthalates, polybrominated diphenyl ethers (PBDEs), PFASs, etc. are scarce or missing (Xue et al. 2015; Yadav et al. 2015; Shekhar et al. 2017; Pednekar et al. 2018; Toxics Link 2018a, b; Babu-Rajendran et al. 2018; Ruan et al. 2019; Malik et al. 2021; Mukherjee Das et al. 2022). In this direction, unlike in many developed countries where EDC human biomonitoring programs are well-established, the role of public health institutions has not been essentially explored in monitoring EDCs and their health consequences for the general Indian population. Primarily, the capacity of available Indian public health infrastructure has been mostly deployed to tackle fundamental health issues rather than the complex EDC health emergency.

Beyond HBM data, substance prioritization and risk assessment require data on environmental and food basket contamination. India is home to a vast agro-ecological diversity and is among the world’s top food producers (including packaged food) (Deloitte 2018). Despite the large export potential of the Indian food industry, there is no dedicated and comprehensive routine monitoring program for residues of contaminants in Indian food produce. Poor control of pesticidal residues in Indian food has already caused a rejection of export to the EU (Kumar 2016).

Risk analysis requires a focus on the full life cycle of a substance. There has been no requirement in India for industry or importers to report data on the volumes and uses. While this is likely to be a feature of the new CMSR, covering a few of the identified EDCs, there is a need to fill past and existing gaps. For this, collecting information on chemical stockpiles, import, manufacturing, sector-wise usage, and waste management will be necessary to estimate emissions and exposure scenarios to EDCs. Lack of such information challenges the government’s capability of formulating and effectively implementing chemical regulations and health protection measures in the country (Steindal and Grung 2021). With such data scarcity, the policymakers will have to rely on international data, which may have limited relevance to the conditions in India. Furthermore, even though consultations are carried out when establishing new regulations, data scarcity will restrict key stakeholders such as civil society and independent, non-governmental bodies from participating constructively in improving current policy and management systems. Consequently, the absence of adequate and publicly available data on EDCs impedes the design of effective and targeted chemical policies and management practices that are relevant to the Indian situation.

### Fragmented Chemical Regulations

With the expansion of the global chemical market, chemical manufacturing and processing activities, which were once centered mainly in the developed industrialized countries, have substantially relocated into the developing countries over the last two decades (UNEP 2019). This combined with increasing purchasing power and an expanding domestic market and in a context of weak environmental and chemical regulation and management, has contributed to increased penetration and use of chemical-intensive products (e.g. personal care products, textile, electronics, building material, toys, processed food, etc.) into the markets of many developing countries (UNEP 2012, 2019), possibly shifting exposure patterns towards areas hosting particularly vulnerable populations.

In India, there are dozens of regulations that directly or indirectly deal with various aspects of managing toxic chemicals at different stages of manufacturing, use, and trade, with a debatable level of efficacy (Sharma et al. 2014b). They are generally framed through an “ad hoc” approach through an “implicit framework” where the chemical assessment and risk management are activated only after advocation based on strong evidence of adverse effects, and whereby the main drivers of exposure are often only partially managed. For instance, to protect children from the adverse health effects of BPA, the Bureau of Indian Standards (BIS) prohibits BPA use in feeding bottles for babies (Mahamuni and Shrinithivihahshini 2017). On the other hand, there are no measures for BPA content in toys, baby-care products, and food and beverage cans, which may contribute to BPA exposure in infants or growing children (Snoj Tratnik et al. 2019).

The existing Indian chemical management regulations policy framework can be divided into four domains: environmental protection, consumer safety, occupational health and safety, and commerce (manufacture, storage, and trade) (Table 1). These include more than a dozen Acts or Rules. None of these regulations specifically addresses or includes a definition of EDCs. Clear frames for prioritizing a substance as an EDC are missing in Indian regulation, although endocrine disruption is suggested as a hazard category in the upcoming CMSR.

**Table 1:** Current Indian regulations relevant for managing EDCs

Regulatory domain	Implementation Area	Relevant Indian regulations
Environment	Water	The Water (Prevention and Control of Pollution) Act 1974 and Rules 1975; the Environment (Protection) Act and Rules 1986
	Soil	The Environment (Protection) Act and Rules 1986
	Air	The Air (Prevention and Control of Pollution) Act 1981 and Rules 1982; the Environment (Protection) Act and Rules 1986
	Waste	The Hazardous Waste (Management, Handling and Transboundary Movement) Rules 1989; the Bio-Medical Waste (Management and Handling) Rules 1998; the Municipal Solid Waste (Management and Handling) Rules 2000; the E-waste (Management and Handling) Rules 2011; the Batteries (Management and Handling) Rules 2001
Consumer safety	Food packaging and additives	The Food Safety and Standards (Packaging) Regulations 2006
	Consumer products (inc. toys etc.)	The Consumer Protection Act 1986; the Public Liability Insurance Act and Rules 1991
Occupational health and safety	Workplace safety	The Chemical Accidents (Emergency Planning, Preparedness, and Response) Rules 1996; the Factories Act 1948
Manufacturing, consumption, and trade	Import	The Manufacture, Storage, and Import of Hazardous Chemical Rules 1989; the Customs Act 1962; the Merchant Shipping Act 1958; the Indian Ports Act 1958
	Chemical related regulations	The Insecticides Act 1968 and Rules 1971; the Pesticides Management Act 2020; the Indian Drugs and Cosmetic Act 1940; Central Insecticide Board and Registration Committee’s guidelines for biocides

The implementation of these acts and rules tends to be mainly based on adopting safety thresholds established in other countries, which may not be adequate for the local context, due to several reasons (population physiology, consumption and exposure patterns, exposure conditions, susceptibility of vulnerable groups, etc.) (James-Todd et al. 2016; Ruiz et al. 2018a; Hass et al. 2019; La Merrill et al. 2020).

Several regulatory and implementing institutions are responsible for India’s fragmented chemical policy frame at the national, state, and local levels, each concerning and covering various parts of overall chemical management. This complexity leads to a lack of integration, unclear jurisdictions, and grey areas resulting in delayed or incomplete implementation and cost overrun. For example, in the Kodaikanal mercury poisoning case, where several tonnes of mercury from a mercury-containing thermometer factory were illegally dumped in a forest causing intoxication of workers and locals, conflicting judgments on health impacts were given by the Ministry of Labour and Employment and a five-member committee constituted by the Madras High Court. Similarly, after the endosulfan tragedy in Kasargod in Kerala, whereby rural workers were deliberately and systematically exposed to the regular aerial spray of endosulfan over two decades, the State government prohibited such a practice in 2005. Yet, the use of endosulfan continued in other parts of India until the nationwide ban imposed in 2011 by an interim order of the Supreme Court of India (Dileep Kumar and Jayakumar 2019).

Fragmentation of regulation and jurisdiction may also trigger lax implementation and enforcement of law. For instance, in 2016 The Ministry of Environment, Forest, and Climate Change (MoEFCC) passed the notification to ban the use of lead (a toxic metal with EDC characteristics) in paints exceeding 90 ppm. However, recent studies have found alarmingly high levels of lead (up to 4 orders of magnitude higher than the legal threshold) in paints from different manufacturers (Toxics Link 2018c, 2019a). Similarly, in 2015, BPA was banned as a plastic additive in baby feeding bottles but studies have shown that this substance is still present in bottles labeled as BPA free (Toxics Link 2019b).

Inefficacy of risk communication to societal stakeholders and the general population also represents a serious hindrance to risk management in India, where a large segment of producers, vendors, and consumers are not recipients of information on the chemical risk inherent to the products they handle, and on the related regulatory provisions inherent to trade, use and waste handling (Tarannum et al. 2018). Moreover, the information on existing chemical management regulations that is publicly available and the corresponding compliance with such regulations may at times be too complex, extensive, and overwhelming, thereby making it inaccessible to these actors (Bhaduri and Sharma 2014; Jain 2020). In such a case, the influence and capacity of public opinion and civil society in shaping environmental protection in general and EDC management in specific will be impeded (Kala et al. 2020; Islam et al. 2021).

The CMSR under development in India has the ambition to face these challenges and lift chemical management and health protection (including from EDCs) to the highest international standard, which is a challenging task. The CMSR will set a more comprehensive list of priority substances harvesting from existing international inventories, including the list of restricted substances or the candidate list of Substances of Very High Concern under REACH. It will foresee a registration process for substances produced or imported into the country based on the classifications of the United Nations Globally Harmonized System of Classification and Labelling of Chemicals (UNGHS). Notably, the current draft list of priority substances does not yet include many priority EDCs such as PBDEs, PFASs, diethyl-meta-toluamide (DEET), or synthetic hormones such as progesterone which have been prioritized in chemical regulations in the EU and the USA (Gore et al. 2015).

### Lacking Risk Analysis Capacity in India

There are several institutions currently engaged in actions for chemical management in India. The CSIR-National Environmental Engineering Institute (NEERI) in India conducts research and development (R&D) studies for solving environmental pollution problems, including those involving some of the EDCs. The institute is also the regional centre of the Stockholm Convention for capacity building and technology transfer for managing POPs in the Asian region (CSIR_NEERI 2019). In addition, other laboratories, institutions [e.g., Food Safety and Standards Authority of India (FSSAI), Central Pollution Control Board (CPCB), State Pollution Control Boards (SPCBs)], and university centres produce independent research on the occurrence, effects, and impacts of EDCs. A key challenge is the lack of infrastructure for creating a knowledge management system connecting knowledge and data provided by different actors to a central, independent risk assessment authority. The European Chemical Agency and the European Food Safety Authority (with clear distinctions and jurisdiction) are devoted to this central role in the European risk analysis system. They serve as recipients and coordinating institutions for the work conducted by national authorities in EU member states. From this perspective, establishing knowledge and data flows linking the key actors, including independent experts, is a priority for the establishment of the CMSR.

Disconnected R&D efforts are not ideal for production and necessary synthesis for operational risk analysis. It leads to widening the knowledge transfer gap within various sectors, cooperation between different stakeholders, and the general public. It further impedes unequivocal, trustable, and authoritative risk communication. Lack of adequate financing and of a harmonized R&D system for designing risk analysis infrastructure have traditionally been major limitations in India in the chemical management sector. The Indian government allocated to the Environment ministry (MoEFCC), $385 million in 2021 compared to $295 million in 2011 (*1 USD* = 74.46 INR), not even compensating for the inflation rate that ranged between 3 and 11% over these years. A major portion of this budget is assigned to managing basic environmental issues like air quality management in Indian cities, National Mission on Green India, wildlife conservation, National Coastal Mission, etc. (Ministry of Finance; McKinsey Global Institute 2020). At the same time, the financial contribution of the private sector to overall environmental management is also minimal. It concentrates on sectors where direct and immediate financial returns are foreseen such as in the sustainable energy and waste management sectors (GIZ 2015).

If confronted with the need for a modern and comprehensive risk-analysis-based chemical management, the Indian institutional and infrastructural system appears poorly adequate. In this context, the risk management area is the most deficient. In the waste management sector, for instance, an estimated 90% of the waste generated in India is dumped in public spaces instead of being disposed of at engineered landfill sites (Kumar et al. 2017). Even in large metropolitan cities like Delhi, waste-to-energy (WTE) plants and landfill sites are out of capacity and located close to densely populated residential areas (Kumar et al. 2017; Randhawa et al. 2020; Ahmed et al. 2020). In rural and semi-urban areas, the state of infrastructure for waste management is often worse than in cities, lacking the basic capacity to collect, segregate, and appropriately manage and dispose of, leaving room for open burning and diffuse sources of hazardous substances (including several EDCs) to the environment (Sutar and Gawande 2013; Chakraborty et al. 2019). Besides municipal waste, the agriculture sector accounts for poorly controlled management of plant protection products that can directly drive human exposure. Several currently used pesticides are confirmed or suspected EDCs. The Integrated Pest Management (IPM) adopted by the Ministry of Agriculture and Farmers Welfare (MoAFW) of the Government of India acts as a cardinal principle and main plank of plant protection and aims to minimize environmental contamination and occupational poisoning. However, in practice, the execution of the rules set by the MoAFW on the ground is below the safety standard since indiscriminate use and disposal of chemical pesticides are still serious issues of concern in India (Sharma et al. 2015; Devegappanavar 2020).

India also lacks expertise and experience in remediating designated “contaminated sites”, even though the MoEFCC through the CPCB and SPCBs ensures appropriate actions against the responsible party [Central Pollution Control Board (CPCB) 2020]. There are about 280 contaminated sites in India, including industrial production areas, landfills, dumps, waste storage, and treatment sites, and chemical waste handler and storage sites. This number is remarkably low compared to for instance the figures for Norway (907) and Switzerland (4000) (Federal Office for the Environment FOEN 2021; Miljøstatus 2021). Such a difference likely reflects insufficient monitoring and inventory capacity to correctly identify and characterize pollution hot spots. Contaminated sites in India raise serious issues of multi-faceted health and environmental problems along with environmental injustice as often populations living close to these sites are socio-economically underprivileged or belong to ethnic minorities (Chatham-Stephens et al. 2013).

Finally, investments are required to establish risk communication infrastructures. Risk communication and stakeholder engagement are key for policy implementation and regulatory frame development, assimilation by societal actors, and acceptance. Identification of the institutional actors responsible for risk communication, the definition of transparent and inclusive rules of engagement with stakeholders, and the establishment of an efficient information infrastructure for risk communication are vital steps that India needs to take.

## A Way Forward: Opportunities for Effective EDC Management

### Enhance EDC Monitoring and Prioritization in India

Establishing a comprehensive and nation-wide monitoring and control system for EDCs in environmental (including food) and human samples is the key to addressing baseline exposure, evaluating the effectiveness of chemical management, and addressing an early-stage possible emergency such as those caused by new pollution hotspots. Monitoring should initially prioritize and focus on legacy and priority EDCs, such as those included in international conventions and regulations. In a subsequent phase, it should be directed towards those EDCs that will be prioritized based on newly available and diversified sources of information, such as those provided by the registration process in the future CMSR and results from the application of risk assessment models on these substances. This will lead to calibrating monitoring efforts towards the specific needs of the Indian society. Such a monitoring system will also contribute to the compilation of priority lists of EDCs in the Indian context and help to create knowledge bases that support research aiming to identify EDCs or potential EDCs relevant in India. The system should comprise expertise from environmental, health-related, social, and economic dimensions to tackle multi-directional impacts of EDC pollution and human exposure.

Establishing human cohort studies that integrate exposome analysis, human biomonitoring, and health outcome records is vital to address the link between exposure and health outcomes specifically in the Indian context. These cohorts should adequately include all the different segments of the Indian population, including vulnerable groups. These cohorts should subsequently monitor various indicators of EDC exposure as well the medical history and the physical environment of the target population so that the EDC exposure levels are linked with the health outcomes, disability-adjusted life year (DALY), under the influence of confounding factors such as socio-economic status, etc. Along with developing an EDC monitoring network, it is essential to create a national repository of EDC exposure data from short-term pilot studies and long-term systematic studies and make them available to stakeholders from different sectors.

The development of open data infrastructure should be accompanied by a sufficient data collection capacity to make data available to the national and international research community. Participation of the academic sector can boost India’s capacity of prioritizing risk analysis actions.

### Defragmenting the EDC Regulatory Framework

The upcoming CMSR is expected to enter into force later in 2022 and may represent an important turning point for chemical management in India. The CMSR will provide risk assessment and management of both individual substances, intermediaries, and mixtures. It will require that any substance manufactured, imported, or placed on the market, above 1 tonne per annum, will have to be registered. The registry will provide a basis for further labelling, handling, and evaluating risk to human health and the environment. In cases of unacceptable risk, restrictions or prohibitions may be warranted. Furthermore, the CMSR may provide a novel opportunity to strengthen the existing regulations for effective EDC management in India.

On paper, the introduction of the CMSR addresses several of the challenges described previously for risk analysis in India, for instance, by resolving regulatory and institutional jurisdiction fragmentations and by introducing a prospective risk management approach (in contrast to the current reactive risk management approach). However, an inherent challenge for the success of this new policy is to ensure the capacity for its effective implementation. Therefore, a serious effort for monitoring the enforcement and, when necessary, introduce corrective actions already in an early stage is required. Public access to the CMSR database and data transparency for research purposes and for civil society actors are essential for a well-functioning and a societal relevant registry.

### Developing and Promoting Health-Preserving Consumption Habits Through Risk Communication

Simply enforcing regulatory mechanisms such as prohibitions or restrictions of EDCs and products containing EDCs may not be the most effective way to rapidly lower EDC pressure on public health. The other avenue is through effective and inclusive risk communication to stakeholders and the public, provided by an independent, trustable, and authoritative institutional voice.

Considering that the major source of exposure to EDCs in the general population is through diet, public awareness-raising, behavioural change, and responsible consumption can be important contributing factors in reducing EDC exposure and the risk of related health consequences. In several developed countries, there are both institutional and independent programs to promote the consumption of organically grown products, better dietary choices, or safer articles (including among others food contact materials), also supported by science communication organizations such as the Food Packaging Forum Foundation (https://www.foodpackagingforum.org/) (Melovic et al. 2020). Currently, North America and Europe account for 90% of the global demand for organic food (Sahota 2019). On the other hand, the potential scope of behavioural change and responsible consumption for minimizing EDC exposure in developing countries like India has not been fully explored. Unlike developed countries, traditional lifestyles, farming, and food in many developing economies are still not entirely replaced by modern industrially processed food production systems and thus presents greater opportunities for the implementation of strategies for responsible consumption and reducing number of chemicals in consumer products (Lee and You 2020; Fenner and Scheringer 2021).

The promotion of organic farming in India started very recently in 2014–15 under the National Mission for Sustainable Agriculture by the MoAFW, which shows that India’s organic farming land comprises only about 2% of the total agricultural land in the country (Khurana and Kumar 2020). Dedicated programs are still required in raising awareness among consumers, train farmers, and officials responsible for the ground-level implementation of government schemes supporting organic farming, and improvising marketing strategies at retail and e-commerce platforms (Raghuveer Singh et al. 2014; Sondhi 2014).

In addition to the farming processes, food-contact materials (packing) also contribute to EDC content in the food (Geueke et al. 2022). Food contact materials are an important area of food safety and health protection regulation in Europe and beyond. India is estimated to be the world’s third-biggest packaged food market with sales reaching 47 million tonnes in 2020. It is a timely opportunity to formulate and implement clear safety and quality standards for food packaging, incentivize producers and consumers towards sustainable and healthy packaging, and promote investment in the application and R&D of green packaging materials. One important benchmark for regulating EDCs in food-contact material in India can be the implementation of the European Regulation (EC) No 1935/2004, which requires food-contact material to be safe, not to transfer components into food in quantities that could endanger human health, and to be traceable throughout the production chain. The regulation also mandates that food contact material must be authorized by the European Food Safety Authority (EFSA) before being placed on the market, a process that is based on the risk analysis approach.

Food industries should invest in promoting the consumption of healthy and safe food. Regularly monitoring the quality of food products for EDCs and other toxic contents and transparently making these results available for consumers would be essential in that regard. For instance, some of the food brands in developed countries provide information on whether their products (e.g. fish oils) contain any highly toxic chemicals such as mercury, lead, PCBs, etc. In this direction, a new Low-Endocrine-Disruptor (LED) food quality labelling can be adopted to foster the creation of a market niche for safer and healthy food and promote sustainable agro- and food business. Similarly, transparent declaration of toxic content in products such as toys, food containers, cosmetics, etc., which are used by vulnerable populations, should be practiced by the manufacturers, particularly on products sold in local open markets in rural and semi-urban areas. Those vulnerable populations are usually unaware of the EDC exposure and related adverse health impacts. Regulatory authorities should strictly monitor and incentivize such practices of declaring harmful content in products. Furthermore, transparent implementation of these actions has the potential for reducing the risk of rejects in food and feed export and for increasing the international market share of Indian food products in the future.

In the direction of developing and promoting health-preserving consumption habits and minimizing health risk from EDC exposure, there are several areas where, apart from the research and legislative institutions, non-governmental organizations (NGOs) have a notable role. In particular, the foremost role of NGOs in India is foreseen in publicizing the issue of EDCs to the general public, especially those living in rural, semi-urban, and underprivileged societies in megacities, simplifying the complex EDC research findings, and presenting the societal perspective on EDC management to policymakers and researchers. In 2022, 51 million people (about 4% of the total population) in India lived in extreme poverty (The Global Statistics 2022) and can be suspected of disproportionate exposure to EDCs as compared to the population with higher income. This is partially because low family income compels them to reside in proximity of active sources of EDC release including areas in vicinity of industries, e-waste handling sites, municipal waste dumpsites and landfills, open waste burning sites, or in slums. Populations residing in these areas in India have been found with elevated body burdens of EDCs (Devanathan et al. 2012; Eguchi et al. 2012). NGOs can take an active role in educating such communities about EDCs and associated health impacts, and to help them relocate to safer locations and identify ways to minimize EDC exposure.

### Public–Private Partnership

In recent years, voluntary public–private partnerships (PPPs) have come to play an essential role in different sectors contributing toward attaining SDGs globally. In India, the focus of PPP programs has been primarily on the infrastructure, health, and education sectors (Kudtarkar 2020). PPP programs can overcome the public sector’s capital deficit and bring the private sector’s competencies and skills to create and manage infrastructure. As a result, possibilities to extend PPP projects to other sectors are constantly being explored (Dolla and Laishram 2020). In recent years, increased involvement of PPP has been observed in various infrastructural projects related to municipal solid waste management in India, in some ways contributing to managing EDCs and other chemical pollutants (Yeboah-Assiamah et al. 2017; Pinupolu and Kommineni 2020). Nevertheless, there is no direct involvement of PPP in EDC management in India, even though there are plenty of opportunities available to support sustainable production and consumption through PPP models. For instance, efficient PPP models can be used to ensure high-quality infrastructure in the food processing sector in India and minimize trade barriers to meet the growing demand for healthy and local food (KPMG 2021). In the same context, the involvement of PPP models is a timely means for modernizing the Indian agricultural sector through building market infrastructure, knowledge, and technology transfer to farmers on sustainable production, etc. (Rankin and Rizzo 2016). In addition, PPP models can help effective adoption of green chemistry metrics in industrial sectors, including the pharmaceuticals and personal care products (PPCP) industry that relies heavily on emerging and priority EDCs, and not least with the important task of developing safer chemical substitutes to EDCs and other chemicals with hazardous properties (Ruiz Sierra et al. 2021). Preliminary steps in this direction are to identify and determine the EDCs for whose management PPP may be a suitable response and accordingly develop PPP supporting capacity-building activities. Overall, there are multidimensional opportunities for using PPP models in minimizing EDC exposure and pollution in India, and their effectiveness will rely on various factors including adequate openness, transparency, monitoring, and effective stakeholder engagement. However, it is pivotal that any such PPP should be framed under clear conditions of accountability, transparency, inclusiveness, and above all be inspired/driven by an independent and objective risk assessment and risk communication.

### Take Account of Lessons from Developed Countries

Rapid growth is expected in the Indian chemical industry over the present decade, with increases in sales from $178 billion in 2020 to $300 billion by 2025 alone (US Department of Trade 2022). The CMSR will provide a crucial framework for exposure at the workplace and of the general public, as it is estimated that 70% of the chemical production is for use within India. The US and EU represent important case studies in effective (EU) and less effective (US) approaches to regulating this growing industry. A risk-based framework (US) has multiple limitations in its capacity to protect public health, while the hazard-based approach in the EU is superior in multiple respects. Researchers have even acknowledged that despite decades of chemical legislation, the chemical governance in the US has not been really successful in protecting human and environmental health (Chiapella et al. 2019). A major issue is that data are lacking to support risk-based approaches, hampering other regulatory actions (Vandenberg et al. 2017). In addition, the lag from identifying new exposures to completing human studies of effects is even more problematic (Kassotis et al. 2020). Because non-monotonic exposure–response relationships exist for many EDCs (Birnbaum 2012; Vandenberg et al. 2012), it is difficult to extrapolate from doses that cause harm to lower doses that are safe (Birnbaum 2012).

If a risk-based regulatory approach prevails in India, it is especially crucial for decision-makers to know how chemicals are being used, and to develop robust biomonitoring programs so that exposures can be evaluated for possible mitigation. Researchers have proposed an International Agency for Research in Endocrine Disruptors to fill data gaps, which describes the state of the evidence for the effects of chemicals on the endocrine system, applying principles similar to those used in systematic reviews (Vandenberg et al. 2016). The agency should delineate processes to establish globally harmonized criteria and mechanisms for identifying EDCs, which can be a substantial support (both in terms of saving financial and intellectual resources) towards managing EDCs in developing and lower- and middle-income countries. Together with advances and acceleration in testing of new and existing chemicals for EDCs, the policy framework in India can become more evidence-based and serve as a model for emerging economies with less capacity for oversight. It is extremely important that India, while strengthening its domestic science basis, also lean on international science and data on EDC research. The resources that the EU has spent over the last decade for developing its EDC framework (still in progress) are tremendous. Not leaning on the experiences (for example of EU and USA) would imply that India—with its emerging economy and infrastructure—would have to invest a substantial cost to reach the same level of action towards EDC management. In the direction of strengthening India’s EDC management actions, both Indian (relevant) data and international experiences/data, without undermining one of them, should go hand on hand.

## Conclusion

EDC pollution in India and several other developing countries is a prime concern in managing EDCs and attaining the SDGs globally. EDC pollution in India has not been as comprehensively and systematically documented as in many developed countries. However, this does not imply that EDC pollution and its health and economic consequences in India are any less severe than those in developed countries. The amplitude of health and economic implications of EDC pollution in India is compounded by other primary health and environmental issues. Considering the need to protect the health of more than 1.3 billion people and the uniquely rich biodiversity in India, a new multifaceted approach is required. Instead of fully relying on the regulations, this approach requires actions upon various aspects of overall chemical management. Foremost, the new chemical-focused regulation, the CMSR, should regulate chemicals (and their products) also based on EDC criteria, and not only based on criteria for persistence (including “very persistent”) and bioaccumulative (including “very bioaccumulative”) and toxicity or for carcinogenicity, mutagenicity, and toxicity for reproduction (CMR). Second, to complement the new chemical-focused regulation, emphasis should be placed on building capacity and infrastructure for comprehensive and systematic monitoring of EDCs, and developing and promoting health-preserving and safe consumption behaviour to minimize EDC exposure. Furthermore, the option of public–private partnerships should be explored for managing sectors that directly or indirectly contribute towards EDC pollution and exposure in India including waste management, green chemicals, organic farming, and food production. Such an ambitious approach can be effectively realized with a transparent involvement of different sectors such as science, policy, governance, NGO, and the private sector. India can take advantage of developed countries’ experience in managing EDCs and set an example for developing countries.

## Data Availability

Data availability statemen
